# Neurodevelopment at 24 months corrected age in extremely preterm infants treated with dexamethasone alternatives during the late postnatal period: a cohort study

**DOI:** 10.1007/s00431-023-05319-z

**Published:** 2023-11-13

**Authors:** Nathalie Melan, Pierre Pradat, Isabelle Godbert, Blandine Pastor-Diez, Eliane Basson, Jean-Charles Picaud

**Affiliations:** 1 Department of Neonatology, Hôpital de La Croix-Rousse, Hospices Civils de Lyon, 69004 Lyon, France; 2 Centre for Clinical Research, Hôpital de La Croix-Rousse, Hospices Civils de Lyon, 69004 Lyon, France; 3 CarMen Laboratory, INSERM, INRA, Université Claude Bernard Lyon 1, Pierre-Bénite, 69310 Lyon, France

**Keywords:** Neurological outcome, Glucocorticoids, Bronchopulmonary dysplasia, Cerebral palsy, Postnatal steroids

## Abstract

The administration of dexamethasone has been associated with suboptimal neurodevelopment. We aimed to compare the development of extremely premature infants treated or not with alternatives to dexamethasone: betamethasone, hydrocortisone hemisuccinate. This retrospective cohort study included infants born before 29 weeks of gestational age, treated or not with late (day ≥ 7) postnatal steroids (betamethasone, hydrocortisone hemisuccinate). The neurodevelopment outcome was evaluated at 24 months corrected age, after adjustment on comorbidities of extreme prematurity. In order to analyse their overall development, data about growth and respiratory outcomes were collected. Among the 192 infants included, 59 (30.7%) received postnatal steroids. Suboptimal neurodevelopment concerned 37/59 (62.7%) postnatal steroid-treated and 43/133 (38.1%; p = 0.002) untreated infants. However, in multivariable analysis, only severe neonatal morbidity (p = 0.007) and male gender (p = 0.027) were associated with suboptimal neurodevelopment outcome at 24 months.

*  Conclusions*: Betamethasone or hydrocortisone hemisuccinate treatment was not an independent risk for suboptimal neurological development, growth and respiratory outcomes assessed at 24 months corrected age in extremely premature infants.

*  Registration number*: The study was registered on the ClinicalTrials.gov register: NCT05055193.

**Table:** 

**What is Known:** *• Late postnatal steroids are used to treat bronchopulmonary dysplasia* *• Meta-analyses warned against the neurological risk of dexamethasone use during neonatal period. Early or late hydrocortisone hemisuccinate has been evaluated in multiple studies, none of which have reported an adverse effect on neurodevelopment at least to 2 years. Data about the use of betamethasone are scarce.*	
**What is New:** *• The risk of suboptimal neurodevelopment was higher among extremely premature infants who received postnatal steroids when compared to those who did not.* *• Betamethasone and hydrocortisone hemisuccinate treatment was not an independent risk factor for suboptimal neurodevelopment at 24 months corrected age.*

**Supplementary Information:**

The online version contains supplementary material available at 10.1007/s00431-023-05319-z.

## Introduction

Postnatal steroids (PNS) are administered to a mean 13% (range: 3.1% to 49.4%) of European infants born before 30 weeks of gestational age (GA) for bronchopulmonary dysplasia (BPD) [1]. There are nonetheless huge regional variations in the proportion of these infants who are treated as well as in the PNS used [1]. The efficacy and tolerance data of PNS almost exclusively relate to dexamethasone (DXM) [1, 2]. Suboptimal neurodevelopment has been associated with its use at low or high doses, and in early (less than 7 days of life) or late (more than 7 days) postnatal periods [3, 4]. Subsequently, hydrocortisone hemisuccinate (HCHS) and betamethasone (BTM) have been more widely used [1, 5]. While no neurodevelopmental risk related to HCHS at 2 years of age has been seen in multiple studies, evidence of neurodevelopmental effects at school age remain insufficient [6], and there is little published data available regarding BTM [3, 7]. Furthermore, glucocorticoids induce impaired alveolar development and postnatal growth [8, 9]. As a consequence, use of PNS is recommended only to help with extubation after the first 3 weeks of life for very premature infants dependent on assisted ventilation, and the recommended products in France are HCHS and BTM [5, 10]. However, the post-discharge consequences of these practices in extremely preterm infants (EPIs) are not well known.

We aimed to assess the impact of alternatives to post-natal DXM on neurodevelopment outcomes at 24 months corrected age (CA) in EPIs.

## Material and methods

### Study design

Retrospective and single-centre cohort study.

### Population

From 01/01/2013 to 31/12/2016, all EPIs born before 29 weeks GA, admitted to the neonatal intensive care unit of the *Hôpital de la Croix-Rousse* (Lyon, France), during at least the first 30 days of life, and alive at 24 months CA were eligible for inclusion; infants with congenital heart, lung, or brain malformation, neuromuscular disease, or genetic disorder were excluded.

### Postnatal steroid regimen

**Fig. 1:**
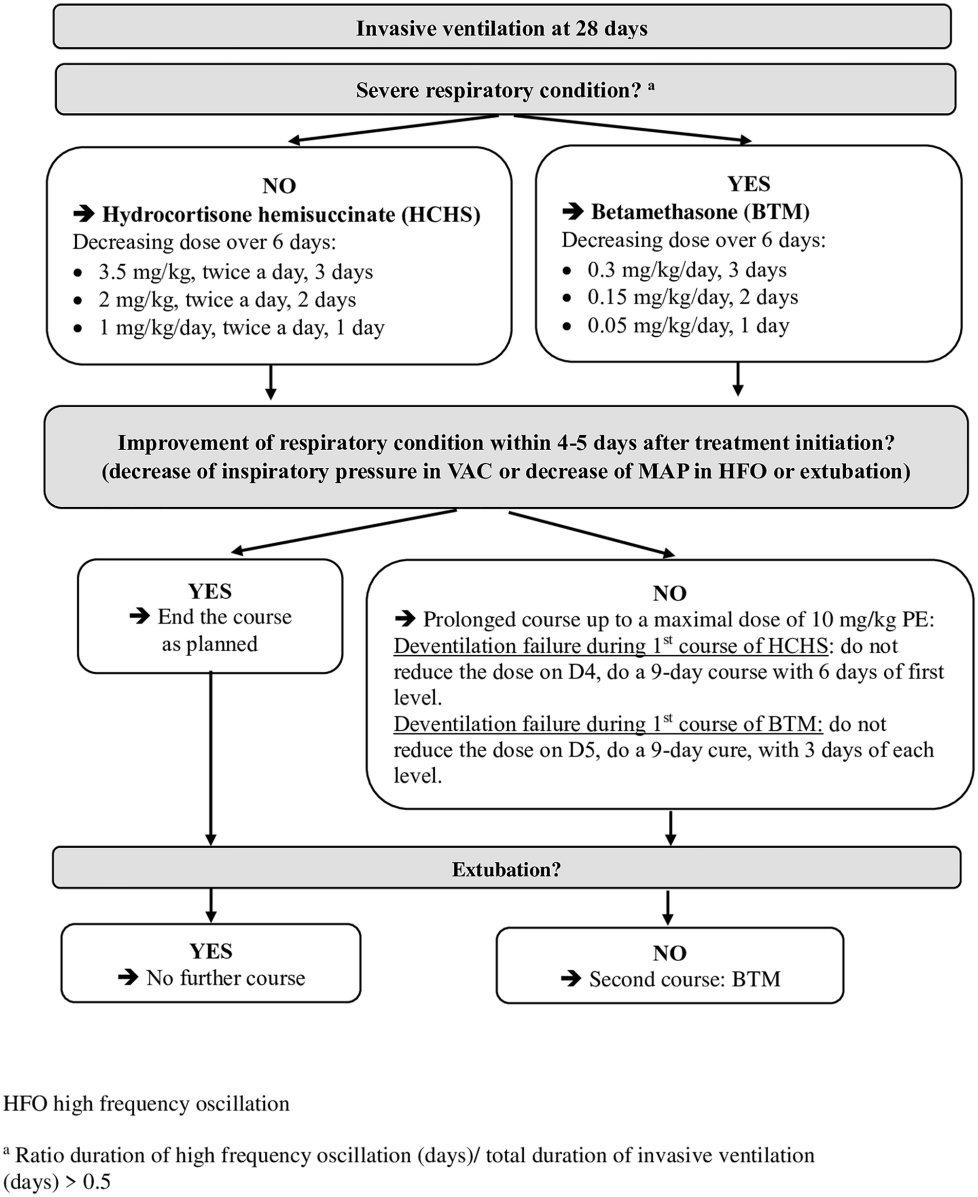
Postnatal steroid treatment protocol

In our unit, conventional mechanical ventilation is the first-line method for invasive ventilation. When lung recruitment is needed (FiO2 > 60% and/or pCO2 > 8.5 kPa, despite ventilation with a maximum inspiratory pressure at 18 cmH2O and a maximum frequency at 50/min in EPIs), high frequency oscillation (HFO) ventilation is used. The transition to conventional mechanical ventilation is done as soon as possible. Postnatal steroid treatment was initiated in the event of failure to wean off assisted ventilation during the late postnatal period (≥ 7 days) [5]. DXM is no longer used in our unit due to the associated neurological risks. BTM (betamethasone 0.05%, oral solution in drops, Laboratoire Arrow Génériques, Lyon, France) or HCHS (hydrocortisone 100 mg, Serb Laboratories, Paris, France) were used according to the severity of the respiratory condition [11] (Fig. 1). Duration of high frequency oscillation was chosen as parameter reflecting respiratory severity, after a study carried out in the department [11].

### Collected parameters

The variations in weight, crown-heel length, and head circumference (HC) were collected, and Z-scores were calculated [12]. Severe and moderate intrauterine growth restriction (IUGR) were defined by a birth weight Z-score < -2 standard deviations (SD; severe IUGR) and < -1 SD (moderate IUGR) [12]. Extra-uterine growth restriction (EUGR) was considered present when body weight was below the 10^th^ percentile (i.e. Z-score < -1.28 SD) at 36 weeks GA [13]. We recorded the duration of invasive and non-invasive ventilation, as well as other neonatal complications: necrotising enterocolitis grade ≥ 2, intraventricular haemorrhage grade ≥ 3, periventricular leukomalacia, and severe retinopathy of prematurity (ROP; stage 2 plus, stage 3, or treatment with laser or anti-VEGF). Severe neonatal morbidity was considered present when at least one of these morbidities was present. We collected the duration of antibiotic therapy. Two days after the beginning of antibiotic therapy for suspected infection, the diagnosis of infection was confirmed collectively, according to clinical course and laboratory results, to decide prolonged antibiotic therapy. At 36 weeks GA, we classified BPD as mild, moderate, or severe [14].

In order to perform a neurodevelopment assessment, we considered the data collected during the systematic follow-up of preterm infants at 24 months CA. The neurodevelopment evaluation was based on the Amiel-Tison neurological examination, revised Brunet-Lézine test, as well as hearing and vision tests [15, 16]. The neurological examination was performed by a paediatrician dedicated to follow-up who had access to the patient’s file and therefore was not masked to administration of steroids. The revised Brunet-Lézine test was used as it is adapted to the early assessment of psychomotor development. It yields a global developmental quotient (DQ) with a mean of 100 and a standard deviation (SD) of 15. DQ is calculated from evaluation of 4 main domains: posture, language, sociability, and coordination. The test was dispensed by a psychologist blinded to the receipt of steroids and cognitive deficit was classified as severe for a DQ ≤ 70, moderate for a DQ > 70 and < 85, and absent for a DQ ≥ 85 [16]. Vision and hearing exams were performed by a specialist blinded to the administration of steroids. Hearing loss was considered as mild or moderate when it was ≥ 20 and ≤ 70 dB, and severe when it was ≥ 71 db.

We also calculated the weight, length, and HC Z-scores-for-age (WHO Anthro software, version 3.2.2) [17]. We collected the presence or absence of hospital admission for wheezing, bronchiolitis, or pneumopathy within the 12 months preceding 24 months CA.

### Main outcome criteria

We considered that neurodevelopment outcome at 24 months CA was suboptimal when there was at least one of the following: abnormal neurological examination, DQ < 85, cerebral palsy, severe hearing loss, unilateral or bilateral blindness, or refractive error.

### Secondary outcome criteria

In order to perform an overall assessment, we considered suboptimal growth when weight, length, or HC Z-scores were lower than -2 SD at 24 months CA and suboptimal respiratory outcome when there was hospital admission for wheezing, bronchiolitis, or pneumopathy within the 12 months preceding 24 months CA. Suboptimal overall development at 24 months CA was defined as the presence of suboptimal neurodevelopment, or suboptimal growth, or suboptimal respiratory outcomes.

### Statistical analyses

We analysed the outcome at 24 months CA of EPIs who received PNS compared to those who did not. Categorical variables were expressed as count (percentage) and continuous variables as median [interquartile range, IQR]. For categorical variables, comparisons between groups were performed using a Chi-squared test or the Fisher exact test, as appropriate. For continuous variables, comparisons between groups were performed using the t-test in case of normal distribution and sample size > 30 in each group, or using the non-parametric Mann–Whitney U test otherwise. For each continuous variable, normality was tested using the Shapiro–Wilk test. A uni- and then multivariable logistic regression analysis was carried out in order to identify factors potentially associated with suboptimal height-weight, neurosensory, or respiratory development at 24 months CA. Due to the limited number of infants with suboptimal development, only variables for which the p-value was < 0.05 in univariable analysis or known or suspected of being associated with the endpoint were included in the multivariable analysis. Variables included in the multivariable analysis on impaired neurodevelopment at 24 months CA were: administration of PNS, sex, duration of assisted ventilation, duration of antibiotic therapy, and severe neonatal morbidity. The results of this analysis were expressed as odds ratios (OR) and 95% confidence interval [95% CI]. Variance inflation factors (VIF) were calculated to test multicollinearity among the independent variables in the multivariable model. The VIF estimates how much the variance of a regression coefficient is inflated due to multicollinearity in the model. VIF starts at 1 and has no upper limit. VIF values exceeding 5 or 10 indicate high multicollinearity between a specific independent variable and the others. All tests were two-sided, a p-value < 0.05 was considered as statistically significant. All analyses were performed using the R software (R Foundation for Statistical Computing, Vienna, Austria).

### Ethics

According to French regulations regarding clinical research, the study was approved by the institutional ethics committee of the *Hospices Civils de Lyon* (IRB 00013204). The encryption and storage of data was registered with the *Commission nationale de l’informatique et des libertés* (CNIL) under the number 21_5613.

## Results

**Fig. 2:**
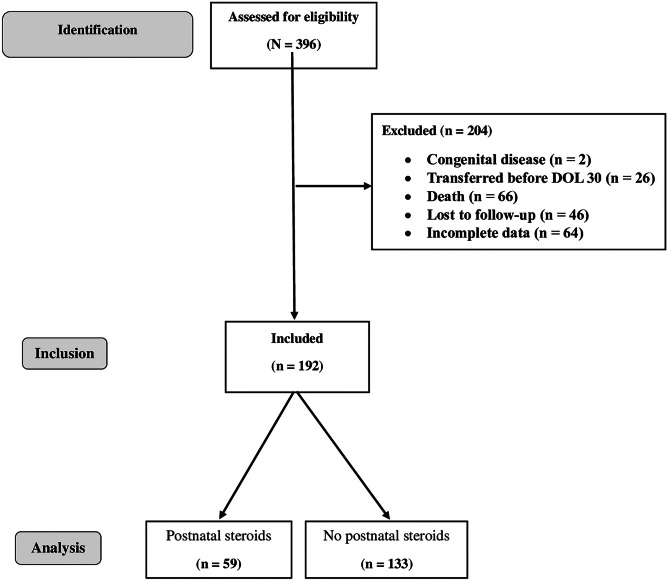
Study flow-chart (DOL, days of life)

During the study period, 396 EPIs were born in the study centre; among them, 192 were included in the present study (Fig. 2), their characteristics are presented in Table 1. Characteristics of included and non-included patients were not significantly different (data not shown). There were 108 infants with severe-to-moderate BPD. Most infants had moderate BPD (72/108), and infants with severe BPD represented 18.8% (36/192) of the population. A total of 59/192 (30.7%) patients received PNS. The median cumulative dose was 8.3 mg/kg prednisolone equivalent, the maximal dose (63 mg/kg prednisone equivalent) was administered to a single patient, and the maximal duration was 45 days (one patient).

**Table 1:** Perinatal characteristics and neonatal morbidity of the 192 extremely premature infants treated or not with postnatal steroids

	**PNS** **N = 59**	**No PNS** **N = 133**	**p**
**Pregnancy and delivery**			
Chorioamnionitis^a^	5 (8.5)	13 (9.8)	0.776
Antenatal steroids	56 (94.9)	124 (93.2)	0.758
Caesarean section	36 (61.0)	99 (74.4)	0.060
Inborn	53 (89.8)	112 (84.2)	0.301
**Birth**			
Gestational age (weeks)	26.0 [25.2–27.4]	27.4 [26.4–28.1]	< 0.001
Male sex	32 (54.2)	65 (48.9)	0.493
Weight (g)	730 [665–845]	920 [750–1050]	< 0.001
Weight Z-score < -1 SD	15 (25.4)	23 (17.3)	0.192
Weight Z-score < -2 SD	2 (3.4)	3 (2.3)	0.644
Length (cm)	32 [30–34]	35 [33–37]	< 0.001
Length Z-score < -2 SD	5 (8.5)	4 (3.0)	0.136
Head circumference (cm)	23 [22–24]	24 [23–25]	< 0.001
Head circumference Z-score < -2 SD	3 (5.1)	4 (3.0)	0.678
**Respiratory morbidities**			
Hyaline membrane disease	59 (100.0)	122 (91.7)	0.019
Assisted ventilation (days)	14.5 [9.3–21.9]	6.0 [1.3–13.7]	< 0.001
HFO ventilation (days)	18.7 [10.1–25.0]	0.0 [0.0–2.7]	< 0.001
Nasal CPAP (days)	26.3 [16.6–35.2]	18.4 [10.2–26.7]	< 0.001
High-flow nasal cannulas (days)	43.6 [30.8–57.9]	32.2 [24.0–41.7]	< 0.001
Pneumonia	26 (44.1)	16 (12.0)	< 0.001
Moderate or severe BPD	53 (89.8)	55 (42.0)	< 0.001
**Other morbidities**			
Necrotizing enterocolitis ≥ grade 2	2 (3.4)	5 (3.8)	1.000
IVH grade 3/4 or PVL	6 (10.2)	12 (9.1)	0.068
ROP ≥ grade 2 + or treated	4 (6.8)	3 (2.3)	0.205
PDA, medically treated	49 (83.1)	70 (52.6)	< 0.001
Duration of antibiotics (days)	22 [14.0–34.5]	6 [2.0–4.0]	< 0.001
Severe neonatal morbidity ^b^	12 (20.3)	20 (15.0)	0.403
**Postnatal steroids treatment**			
BTM	38/59 (64.4)		
HCHS	19/59 (32.2)		
HCHS and BTM	2/59 (3.4)		
Cumulative dose, mg/kg PE	8.3 [7.6–10.0]		
Cumulative duration, days	6.0 [6.0–9.0]		

Data are expressed as count (percentage) or median [interquartile range]

*PNS* postnatal steroids, *SD* standard deviation, *HFO* high frequency oscillation, *CPAP* continuous positive airway pressure, *BPD* bronchopulmonary dysplasia, *IVH* intraventricular haemorrhage, *PVL* periventricular leukomalacia, *ROP* retinopathy of prematurity, *PDA* patent ductus arteriosus, *BTM* betamethasone, *HCHS* hydrocortisone hemisuccinate, *PE* prednisone equivalent

^a^Typical placental lesion, maternal fever, increased protein C reactive, presence of fetid amniotic fluid

^b^Severe IVH or PVL or necrotising enterocolitis or ROP

**Fig. 3:**
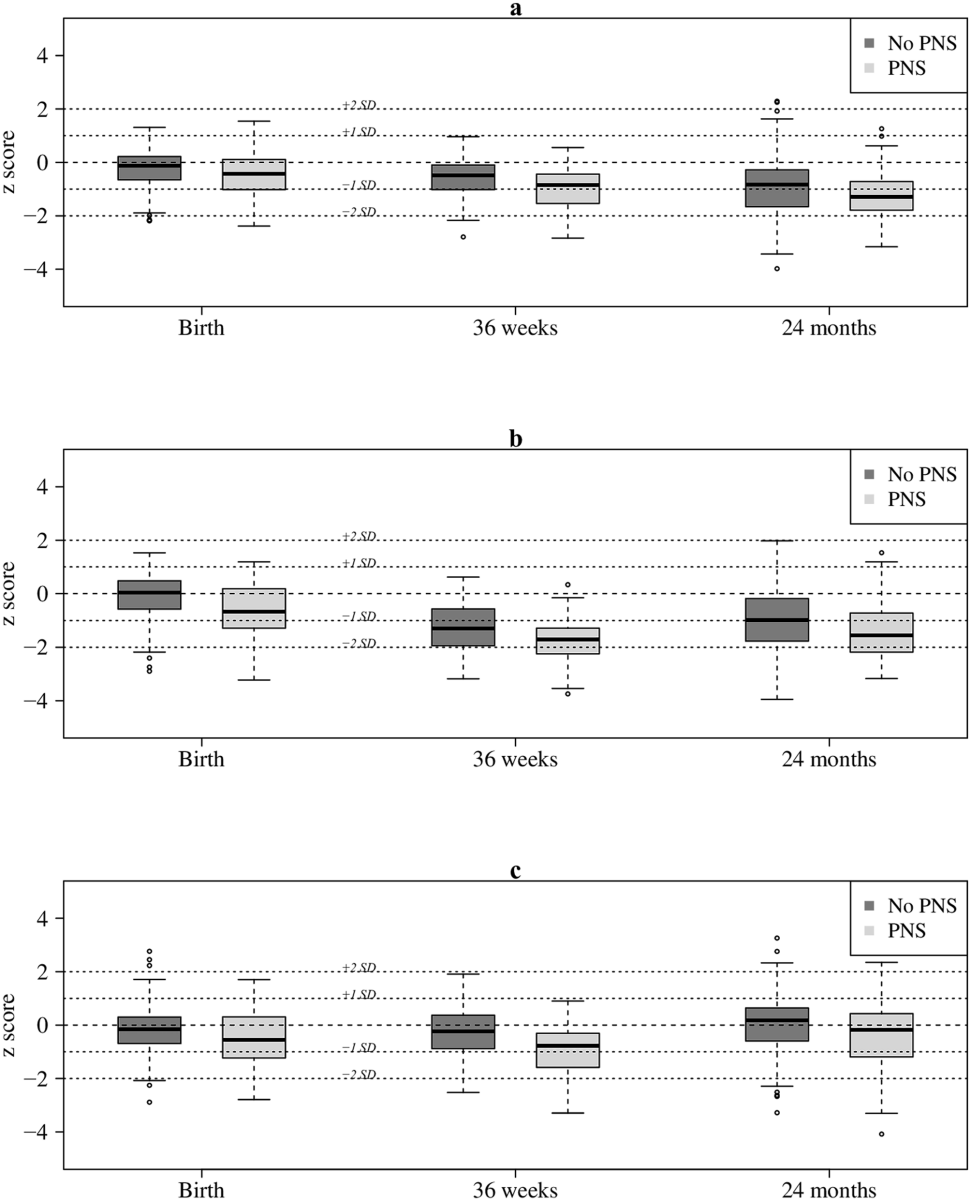
Z-score for gestational age for body weight (**a**), length (**b**), and head circumference (**c**) at birth, 36 weeks postconceptional age and at 24 months corrected age in 192 extremely premature infants treated or not with postnatal steroids. PNS postnatal steroids, SD standard deviation. There was no significant difference between the 2 groups at each time and for each measure

The Z-scores for body weight, length and head circumference were similar in the two groups at birth, 36 weeks GA and at 24 months CA (Fig. 3). At 36 weeks GA, the proportion of infants with EUGR for body weight, length and head circumference was significantly higher in the treated group. There was a deficit in HC growth in the PNS group between birth and 36 weeks GA (delta Z-score: PNS, -0.53 SD; no PNS, + 0.23 SD; p = 0.003), without any significant difference in HC growth between 36 weeks GA and 24 months CA (Supplementary Table 1).

The systematic follow-up of preterm infants was performed at the median [IQR] CA of 24.2 [23.8; 24.8] months. The proportion of infants for whom the clinical neurological examination was abnormal was higher in the PNS group (14/55, 25.5% vs. 17/127, 13.4%; p = 0.047). The proportion of infants with a DQ < 85 was higher in the PNS group (10/46, 21.7% vs. 8/113, 7.1%; p = 0.008; Table 2). Three patients had a DQ < 70, all were in the PNS group. Therefore, suboptimal neurodevelopment was significantly more frequent among treated (20/59, 33.9%) than among untreated infants (23/133, 17.3%; p = 0.008; Table 2).

**Table 2:** Neurodevelopmental outcome at 24 months corrected age (primary study endpoint)

	**PNS** **N = 59**	**No PNS** **N = 133**	**p**
**Neurological outcome**			
Abnormal neurological clinical examination	14/55 (25.5)	17/127 (13.4)	0.047
DQ (Brunet-Lézine) < 85	10/46 (21.7)	8/113 (7.1)	0.008
Cerebral palsy	8/55 (14.5)	12/127 (9.4)	0.313
Hearing impairment	2/58 (3.4)	1/131 (0.8)	0.223
Visual impairment	3/52 (15.1)	5/116 (12.5)	0.681
**Suboptimal neurodevelopment**	20 (33.9)	23 (17.3)	0.008

Data are expressed as count/total (percentage)

*PNS* postnatal steroids, *DQ* developmental quotient

In multivariable logistic regression analysis, the use of PNS was not independently associated with suboptimal neurodevelopment at 24 months CA (OR = 1.04, 95% CI [0.37; 2.95]; p = 0.939). Conversely, severe neonatal morbidity (OR = 4.21, 95% CI [1.48–12.0]; p = 0.007) was an independent predictor of suboptimal neurodevelopment. Although the association did not reach statistical significance, the duration of antibiotic therapy (per additional week) was slightly associated with suboptimal neurodevelopment with a 29% increase in risk for each additional week of antibiotic therapy (OR = 1.29, 95% CI [0.98–1.70]; p = 0.069; Table 3). Female sex (OR = 0.40, 95% CI [0.18–0.90]; p = 0.027) appeared as a protective factor for suboptimal neurodevelopment. VIF scores for administration of PNS, sex, assisted ventilation, duration of antibiotic therapy, and severe morbidity were 1.7, 1.0, 1.9, 1.8, and 1.1, respectively.

**Table 3:** Uni- and multivariable analysis on suboptimal neurodevelopment at 24 months ^a^

	**Univariable analysis**		**Multivariable analysis**	
	**OR [95% CI]**	**p**	**OR [95% CI]**	**p**
Postnatal steroids	2.58 [1.26; 5.29]	0.009	1.04 [0.37–2.95]	0.939
Oligoamnios	1.59 [0.51; 4.95]	0.421	-	-
Chorioamnionitis	0.63 [0.17; 2.29]	0.480	-	-
Antenatal steroids	1.51 [0.31; 7.29]	0.606	-	-
Gestational age (per additional week)	0.90 [0.69; 1.19]	0.462	-	-
Sex (female versus male)	0.43 [0.21; 0.89]	0.024	0.40 [0.18–0.90]	0.027
Weight Z-score < -1 SD	1.59 [0.68; 3.70]	0.285	-	-
Assisted ventilation (per additional week)	1.04 [1.02; 1.06]	< 0.001	1.14 [0.92–1.41]	0.217
Duration of antibiotics (per additional week)	1.06 [1.04; 1.09]	< 0.001	1.29 [0.98; 1.70]	0.069
Pneumonia	2.78 [1.30; 5.98]	0.009	-	-
Severe neonatal morbidity	5.27 [2.17; 12.8]	< 0.001	4.21 [1.48–12.0]	0.007
Moderate or severe BPD	1.49 [0.99; 2.25]	0.055	-	-
Postnatal steroid dose ^b^ (per additional mg/kg)	1.01 [0.96; 1.05]	0.780	-	-
ΔZ-score ^c^ for weight (per 1 SD)	0.52 [0.26; 1.00]	0.051	-	-
ΔZ-score ^c^ for length (per 1 SD)	0.71 [0.42; 1.21]	0.208	-	-
ΔZ-score ^c^ for HC (per 1 SD)	0.77 [0.53; 1.10]	0.150	-	-

*OR* odds ratio, *CI* confidence interval, *SD* standard deviation, *BPD* bronchopulmonary dysplasia, HC head circumference

^a^at least one of the following: abnormal neurological examination, DQ < 85, cerebral palsy, severe hearing loss, unilateral or bilateral blindness, or refractive error

^b^In prednisone equivalent

^c^Difference in Z-score between birth and 36 weeks postmenstrual age

Growth and respiratory outcomes were secondary outcomes. The proportion of patients with HC growth deficit was higher in the PNS group (9/57, 15.8%) compared to the untreated group (8/131, 6.1%; p = 0.033). There was no significant difference in the proportion of infants admitted to hospital for a respiratory event during the second year of life (12/57, 21.1% vs. 20/130, 15.4%; p = 0.724). The suboptimal overall development was significantly more frequent among treated (39/59, 66.1%) than among untreated infants (62/133, 46.6%; p = 0.013; Supplementary Table 2).

The use of PNS was not independently associated with suboptimal overall development at 24 months CA (OR = 0.81, 95% CI [0.31; 2.15]; p = 0.673). Conversely, birth weight Z-score < -1 SD (OR = 4.63, 95% CI [1.81; 11.9]; p = 0.001) was independently associated with suboptimal overall development at 24 months CA. The delta Z-score for weight between birth and 36 weeks GA was a protective factor (OR = 0.48, 95% CI [0.25; 0.92]; p = 0.028; Supplementary Table 3).

## Discussion

The risk of suboptimal neurodevelopment at 24 months CA was higher among EPIs who received alternatives to post-natal DXM, which was expected as these infants were sicker, but PNS using short treatments of BTM or HSHC was not found to be an independent risk factor for suboptimal neurodevelopment. Conversely, male sex, severe neonatal morbidity and to a lesser extent, duration of antibiotic therapy appeared as independent risk factors for suboptimal neurodevelopment.

In preterm infants, particularly in EPIs, neonatal infections have been reported to be associated with suboptimal later neurodevelopment [18–21], which could be explained by the inflammation and hemodynamic changes associated with infections that can damage the white matter [22, 23]. Herein, the duration of antibiotic therapy can be considered as a surrogate of the exposure to neonatal infections as treatment longer than 2 days is collectively decided in our unit, according clinical course and laboratory results; only infants with confirmed infection are therefore exposed to prolonged antibiotic therapy. Although the association between antibiotic therapy and neurodevelopment is slightly above significance, the results of the present study suggest that preventing infection in EPIs may contribute to the protection of the premature brain and reduction of the risk of suboptimal neurodevelopment in EPIs.

Contrary to that previously reported with DXM [3, 4], the results of the present study do not support an impact of alternatives to post-natal DXM on the neurodevelopment at 24 months CA. DXM treatment has been associated with a decrease in cerebral volume related to a decrease in the volume of grey matter and cerebellum [24], which persisted after adjustment on neonatal comorbidities, including BPD [25]. In studies using HCHS, similarly to herein, after the 7^th^ day of life and at doses of 10 to 34 mg/kg/course of prednisone equivalent there was no impaired neurodevelopment at 24 months CA; in addition, there was neither brain volume abnormality at term-equivalent age, or impaired neurodevelopment at school age [6, 26]. Recent studies regarding early HCHS and neurodevelopment at 2 and 5 years of age reported results consistent with the main findings of the present study [27–29]. The impact of BTM on neurological development is poorly known [5]. The effect of steroids on the developing brain can be explained by their pharmacological properties; DXM is a synthetic steroid 25 times more potent than HCHS and its interaction with glucocorticoid receptors in the brain causes adverse neuronal effects on the hippocampus by activating the apoptotic pathway, while HCHS mainly interacts with mineralocorticoid receptors [30]. The 11b-hydroxysteroid dehydrogenase type 2 enzyme is widely expressed in the foetal or preterm brain and metabolises active HCHS into the inactive form, 11-dehydroxysteroid [31]; there is no equivalent enzyme for DXM metabolism in the human brain. In addition, DXM is administered with sodium bisulphite for its preservation, unlike HCHS and BTM, and this excipient is toxic to neuronal cell lines [32].

As prolonged invasive ventilation, administration of PNS, and presence of severe neonatal morbidity are possibly linked, we calculated VIF scores to test multicollinearity among the independent variables. All VIF scores were close to 1, suggesting absence of collinearity in the model.

The absence of association between PNS, as used in the present study, and suboptimal development at 24 months CA is likely because the current preterm population and therapies are different from the eighties and nineties [3, 4]. BTM and HSHC were used for shorter durations, at lower doses, and in a setting where optimised nutritional care has been shown to reduce the risk of postnatal growth failure [33]. Another important point of note is the protective factors identified herein, which seem relevant.

The duration of assisted ventilation, being moderately weight-restricted at birth, and the postnatal weight deficit were independent risk factors of impaired overall development (neurodevelopment, growth, and respiratory outcomes) at 24 months CA. Each additional week of invasive ventilation was associated with a 24% increased risk of suboptimal overall development at 24 months CA although this increased risk did not reach statistical significance (p = 0.060). This possible association could be explained by inflammation, oxidative stress, hemodynamic changes that are related to assisted ventilation and prolonged sedation [34, 35]. In previous studies, it is reported that each additional day of invasive ventilation increases the risk of neuro-developmental impairment at 24 months CA, and brain metrics at term-equivalent age together with postnatal HC growth were both independently associated with the duration of mechanical ventilation and not with HCHS [36, 37]. The results or the present study suggest that the risk–benefit balance between alternatives to post-natal DXM and prolonged assisted ventilation is in favour of PNS treatment aiming at limiting the duration of exposure to mechanical ventilation. In addition, being weight-restricted at birth was a significant and independent risk factor of suboptimal overall development at 24 months herein, which is in agreement with previously published data [38]. IUGR has also been associated with a suboptimal respiratory function at school age, and suboptimal growth at 6 years [39]. The results presented herein therefore suggests that EPIs who are weight-restricted at birth require a specific follow-up of their further development. We also identified weight gain between birth and 36 weeks GA as a protective factor of suboptimal overall development, as previously reported [39]. Herein, postnatal weight gain was close to foetal weight gain during the third trimester in both groups, which suggested the absence of major effect of PNS on growth in this population. However, when considering differences in Z-scores between birth and 36 weeks GA, there was a deficit in both groups, which was slight for body weight and moderate (about 1 SD) for length. For HC, only infants treated with PNS presented a postnatal deficit, while untreated infants had a gain in HC Z-score between birth and 36 weeks. This suggests that it is probably still possible to improve the nutritional care in EPIs, and advocates for a close monitoring of postnatal growth.

A major limitation of the study is the single-centre, retrospective design. Although the results only reflect the experience of an individual centre, the study population was representative of EPIs, as neonatal morbidities were close to that reported in large national cohorts [40]; the findings are therefore likely to be applicable to the population of EPIs admitted to other units. However, the retrospective nature explains the why some patients were not included because of insufficient data. However, there was no significant difference between those included and those not included in the study. Furthermore, the single-centre design prevented us from including a greater number of infants, and the limited number of infants with the outcome of interest (suboptimal neurodevelopment) made it difficult to include a greater number of variables in the multivariable analysis and limited the power to detect risk factors.

In conclusion, the use of BTM or HCHS, alternatives to post-natal DXM, was not associated herein with suboptimal neurodevelopment at 24 months CA in EPIs infants. These rather reassuring results, together with the few published data on this topic, will allow to design prospective randomised studies aimed at comparing DXM and alternative products (BTM, HCHS) in neonatology units.

### Supplementary Information

Below is the link to the electronic supplementary material.

Supplementary file1 (DOCX 28 KB)

Supplementary file2 (DOCX 26 KB)

Supplementary file3 (DOCX 29 KB)

## Data Availability

Data are available upon reasonable request to the corresponding author.

## Abbreviations

BPD: Bronchopulmonary dysplasia
BTM: Betamethasone
CA: Corrected age
DQ: Developmental quotient
DXM: Dexamethasone
EPIs: Extremely preterm infants
EUGR: Extra-uterine growth restriction
GA: Gestational age
HC: Head circumference
HCHS: Hydrocortisone hemisuccinate
HFO: High frequency oscillation
IQR: Interquartile range
IUGR: Intrauterine growth restriction
OR: Odds ratio
PNS: Postnatal steroids
ROP: Retinopathy of prematurity
SD: Standard deviation
VIF: Variance inflation factors
