# Supplementary material for: Neurodevelopment at 24 months corrected age in extremely preterm infants treated with dexamethasone alternatives during the late postnatal period: a cohort study
Source: Eur J Pediatr. 2023 Nov 13;183(2):677–87. doi: 10.1007/s00431-023-05319-z (PMC10912127; doi:10.1007/s00431-023-05319-z)
Supplement: Supplementary file 1 — Supplementary file1 (DOCX 28 KB) [file 431_2023_5319_MOESM1_ESM.docx]

**Supplementary Table 1.** Growth between birth and 36 weeks postconceptional age and between 36 weeks postconceptional age and 24 months corrected age in 192 extremely premature infants treated or not with postnatal steroids.

|  | **PNS**  **N = 59** | **No PNS**  **N = 133** | **p** |
| --- | --- | --- | --- |
| **Gain between birth and 36 weeks GA** |  |  |  |
| Body weight (g/kg/day) | 14.7 [13.3-15.8] | 15.0 [14.1-15.9] | .156 |
| Length (cm/week) | 1.07 [0.94-1.15] | 1.05 [0.88-1.16] | .401 |
| Head circumference (cm/week) | 0.82 [0.73-0.91] | 0.93 [0.79-1.00] | .005 |
| **EUGR** |  |  |  |
| Body weight | 20/58 (34.5) | 21/112 (18.8) | .023 |
| Length | 44/58 (75.9) | 56/112 (50.0) | .001 |
| Head circumference | 19/58 (32.8) | 11/110 (10.0) | .001 |
| **ΔZ-score birth-36 weeks GA** |  |  |  |
| Body weight, SD | -0.30 [-1.04 - 0.04] | -0.24 [-0.65 - -0.01] | .074 |
| Length, SD | -0.99 [-1.56 - -0.68] | -1.06 [-1.51 - -0.63] | .961 |
| Head circumference, SD | -0.53 [-1.19 - 0.21] | 0.23 [-0.51 - 0.59] | .003 |
| **Gain between 36 weeks GA and 24 months CA** | | |  |
| Body weight (g/kg/day) | 10.8 [10.2 - 12.0] | 11.3 [10.0 - 12.5] | .579 |
| Length (cm/week) | 1.66 [1.58 - 1.74] | 1.66 [1.56 - 1.75] | .937 |
| Head circumference (cm/week) | 0.64 [0.62 - 0.69] | 0.63 [0.59 - 0.68] | .235 |
| **ΔZ-score 36 weeks GA - 24 months CA** |  |  |  |
| Body weight, SD | -0.35 [-0.81 - 0.34] | -0.40 [-1.08 - 0.20] | .379 |
| Length, SD | 0.41 [-0.19 - 0.91] | 0.27 [-0.46 - 0.96] | .490 |
| Head circumference, SD | 0.49 [-0.05 - 1.20] | 0.29 [-0.33 - 0.93] | .132 |

GA gestational age, PNS postnatal steroids, SD standard deviation, Δ Z-score difference in Z-score, CA corrected age, EUGR extra-uterine growth restriction (<10^th^ percentile or -1.28SD at 36 weeks GA)

Data expressed as count/total (percentage) and median [interquartile range].
