# Supplementary material for: Neurodevelopment at 24 months corrected age in extremely preterm infants treated with dexamethasone alternatives during the late postnatal period: a cohort study
Source: Eur J Pediatr. 2023 Nov 13;183(2):677–87. doi: 10.1007/s00431-023-05319-z (PMC10912127; doi:10.1007/s00431-023-05319-z)
Supplement: Supplementary file 2 — Supplementary file2 (DOCX 26 KB) [file 431_2023_5319_MOESM2_ESM.docx]

**Supplementary Table 2.** Overall outcome at 24 months corrected age (secondary study endpoint)

|  | **PNS**  **N = 59** | **No PNS**  **N = 133** | **p** |
| --- | --- | --- | --- |
| **Suboptimal neurodevelopment** | 37/59 (62.7) | 43/133 (38.1) | .002 |
| **Growth outcome** |  |  |  |
| Weight Z-score < -2 SD | 9/58 (15.5) | 27/132 (20.5) | .424 |
| Length Z-score < -2 SD | 17/58 (29.3) | 23/132 (18.9) | .064 |
| Head circumference Z-score < -2 SD | 9/57 (15.8) | 8/131 (6.1) | .033 |
| **Respiratory outcome** |  |  |  |
| Hospital admission | 12/57 (21.1) | 20/130 (15.4) | .724 |
| **Suboptimal overall development** | 39 (66.1) | 62 (46.6) | .013 |

PNS postnatal steroids, SD standard deviation

Data are expressed as count/total (percentage).
