# Supplementary material for: Neurodevelopment at 24 months corrected age in extremely preterm infants treated with dexamethasone alternatives during the late postnatal period: a cohort study
Source: Eur J Pediatr. 2023 Nov 13;183(2):677–87. doi: 10.1007/s00431-023-05319-z (PMC10912127; doi:10.1007/s00431-023-05319-z)
Supplement: Supplementary file 3 — Supplementary file3 (DOCX 29 KB) [file 431_2023_5319_MOESM3_ESM.docx]

**Supplementary Table 3.** Univariable and multivariable analysis on the secondary study endpoint: impaired overall development at 24 months corrected age.

|  | **Univariable analysis** | | **Multivariable analysis** | |
| --- | --- | --- | --- | --- |
|  | **OR [95% CI]** | **p** | **OR [95% CI]** | **p** |
| Postnatal steroids | 2.23 [1.18-4.23] | .014 | 0.81 [0.31-2.15] | .673 |
| Oligoamnios | 1.32 [0.48-3.62] | .592 | - | - |
| Chorioamnionitis | 0.70 [0.26-1.85] | .468 | \| - \| - \| \| --- \| --- \| | - |
| Antenatal steroids | 1.12 [0.35-3.60] | .852 | \| - \| - \| \| --- \| --- \| | - |
| Gestational age (per additional week) | 0.85 [0.67- 1.07] | .159 | 1.03 [0.74-1.43] | .861 |
| Sex (Female versus male) | 0.61 [0.34-1.07] | .085 | \| - \| - \| \| --- \| --- \| | - |
| Weight Z-score < -1 SD | 3.67 [1.36-8.27] | .002 | 4.63 [1.81-11.9] | .001 |
| Weight Z-score < -2 SD | 3.71 [0.41-33.80] | .245 | - | - |
| Assisted ventilation (per additional week) | 1.04 [1.02-1.06] | < .001 | 1.24 [0.99-1.56] | .060 |
| Duration of antibiotics (per additional week) | 1.04 [1.01-1.07] | < .001 | 1.09 [.84-1.44] | .512 |
| Pneumonia | 2.42 [1.17-5.01] | .018 | - | - |
| Severe neonatal morbidity | 2.00 [0.85-4.71] | .113 | 1.71 [0.59-4.95] | .324 |
| Moderate or severe BPD | 1.57 [1.13-2.18] | .007 | - | - |
| Postnatal steroids dose ^a^ (per additional mg/kg) | 1.09 [0.98-1.22] | .112 | - | - |
| ΔZ-score ^b^ for weight (per 1 SD) | 0.52 [0.30-0.91] | .021 | 0.48 [0.25-0.92] | .028 |
| ΔZ-score ^b^ for length (per 1 SD) | 0.75 [0.48-1.19] | .225 | - | - |
| ΔZ-score ^b^ for HC (per 1 SD) | 0.87 [0.64-1.19] | .387 | - | - |

OR odds ratio, CI confidence interval, SD standard deviation, BPD bronchopulmonary dysplasia, HC head circumference

^a^ In prednisone equivalent

^b^ Difference in Z-score between birth and 36 weeks postmenstrual age.
